# Enhanced Crystallinity of Triple-Cation Perovskite Film via Doping NH_4_SCN

**DOI:** 10.1186/s11671-019-3134-4

**Published:** 2019-09-02

**Authors:** Ziji Liu, Detao Liu, Hao Chen, Long Ji, Hualin Zheng, Yiding Gu, Feng Wang, Zhi Chen, Shibin Li

**Affiliations:** 1 State Key Laboratory of Electronic Thin Films and Integrated Devices, and School of Optoelectronic Science and Engineering, University of Electronic Science and Technology of China (UESTC), Chengdu, 610054 Sichuan China; 2 Department of Electrical & Computer Engineering, Center for Nanoscale Science & Engineering, University of Kentucky, Lexington, KY 40506 USA

**Keywords:** Perovskite solar cells, NH_4_SCN, Crystalline, Trap-state density

## Abstract

The trap-state density in perovskite films largely determines the photovoltaic performance of perovskite solar cells (PSCs). Increasing the crystal grain size in perovskite films is an effective method to reduce the trap-state density. Here, we have added NH_4_SCN into perovskite precursor solution to obtain perovskite films with an increased crystal grain size. The perovskite with increased crystal grain size shows a much lower trap-state density compared with reference perovskite films, resulting in an improved photovoltaic performance in PSCs. The champion photovoltaic device has achieved a power conversion efficiency of 19.36%. The proposed method may also impact other optoelectronic devices based on perovskite films.

## Introduction

Owing to the excellent optoelectronic properties, the organic-inorganic hybrid metal halide perovskite (OIMHP) has been widely used as the light-harvester material of solar cells. The last certified power conversion efficiency (PCE) of the solar cells based on OIMHP has reached 24.2% [1]. Perovskite solar cells (PSCs) are the most promising solar cells among the third-generation solar cells.

The bandgap of conventional OIMHP films is at the range of 1.5–1.6 eV, and the corresponding theoretical Shockley–Queisser limit efficiency (TS-QLE) is higher than 30% [2–4]. However, the reported highest PCE is much lower than the TS-QLE due to the trap-assisted non-radiative recombination in the perovskite film [5–8]. The trap-assisted non-radiative recombination intensity often depends on the defect density in perovskite films and most of the defects are spread on the surface and boundary of perovskite crystal grains due to the atomic vacancies [7, 9]. Therefore, perovskite films with less crystal grain boundary area contribute to the better photovoltaic performance of PSCs [10–12]. The perovskite films with less crystal grain boundary area can be obtained via increasing the crystal grain size. To enlarge the crystal grain size of perovskite films, various methods have been developed, including the additive engineering [13–15], precursor solvent engineering [16], anti-solvent engineering [17], and procedure optimization [18–20]. Among these methods, the additive engineering is one of the most frequently used methods to the realization of large-crystal-grain-size perovskite films. The additive materials include polymers [21], small organic molecules [15, 22], and inorganic salts [23]. The polymers with special organic groups like carbonyl groups can slow down the crystallization process and enlarge the grain size of the perovskite films [21]. The carbonyl bonds containing lone electron pairs can interact with the Lewis acid PbI_2_ in the precursor solution and the intermediate polymer-PbI_2_ adduct forms. The formation of the adduct retards the crystal growth and improves the crystallinity of the perovskite film. Bi et al. employed poly(methyl methacrylate) (PMMA) as a template to control the crystallization process of perovskite, improving the PCE up to 21.6% [21]. Small organic molecules containing special groups are also used frequently to adjust the crystallinity of perovskite films. The mechanism for improving the crystallinity of perovskite is the same as polymers. To avoid the defect formation, the small organic molecules are required to have suitable energy levels. Zhang et al. used a fused ring electron acceptor material to dope perovskite films. This material improved the crystallinity of perovskite films and increased the PCE of PSCs from 19.6% to 21.7% [22]. The inorganic salts used in perovskite films include Pb(SCN)_2_, KSCN, NaSCN, CdCl_2_, and NiCl_2_ [14, 24, 25]. The SCN^−^ has a larger electronegativity than the I ionic, so SCN^−^ anion is more apt to form ionic bonding with the CH_3_NH_3_^+^ cation than the I^−^ anion. The formed ionic bonding can also retard the crystal growth and increase the crystallinity of perovskite films. When the perovskite was heated at high temperature, the SCN^−^ can escape from perovskite films and the metal ions can be left. The Cd^2+^ and Ni^2+^ in perovskite precursors can change the crystal growth mechanism and improve the crystallinity of perovskite films.

The ammonium thiocyanate (NH_4_SCN) contains SCN^−^ anion, so it can improve the crystallinity of perovskite films [11]. This material in perovskite films can be decomposed into HSCN and NH_3_ when samples are heated on hotpot. Therefore, no residual of NH_4_SCN will be left in perovskite films and defects induced by the introduction of NH_4_SCN will not appear. From the above analysis, NH_4_SCN is an effective additive for improving the crystallinity of perovskite films, which has been proved by Zhang et al. [26]. Chen’s group has used NH_4_SCN to enhance the crystallinity of FAPbI_3_ films and form the vertically oriented 2D-layered perovskite films [27–29]. Ning’s group introduced NH_4_SCN into tin-based perovskite films to manipulate the crystal growth process, which improved the photovoltaic performance and stability of tin-based PSCs [30].

Here, the NH_4_SCN was employed to control the crystallinity of triple-cation perovskite films. The NH_4_SCN can increase the crystal grain size and reduce the boundary area in perovskite films, inducing a lower trap-state density. The lower trap-state density attributes to the longer charge lifetime and higher photovoltaic performance of PSCs. The PCE of PSCs has been improved from 17.24% to 19.36%.

## Method

### Materials

All of the materials were purchased from Ying Kou You Xuan Trade Co., Ltd., if not specified. The PbI_2_, tris(2-(1H-pyrazol-1-yl)-4-tert-butylpyridine)-cobalt(III) bis(trifluoromethylsulphonyl)imide (FK209), PEDOT:PSS, and FAI were purchased from Xi’an Polymer Light Technology Cory. CsI, dimethylformamide (DMF), and dimethyl sulfoxide (DMSO) were purchased from Sigma-Aldrich Corp. The SnO_2_ nanoparticle colloidal solution was purchased from Alfa Aesar.

The perovskite solution was prepared as follows: 507 mg PbI_2_, 73.4 mg PbBr_2_, 172 mg FAI and 22.4 mg MABr was dissolved into 1 mL solvent mixture (V(DMSO):V(DMF) = 3:7) to prepare the solution 1. Then, 52-μL CsI solution (390 mg in l mL DMSO) was added into the solution 1 and then the final solution was stirred for 2 h. For the NH4SCN-doped perovskite solutions, different mass of NH4SCN was added into the prepared perovskite solutions directly and the final solutions were stirred for 2 h. The HTL solution was prepared by dissolving 72.3 mg (2,29,7,79-tetrakis(*N*,*N*-di-p-methoxyphenylamine)-9,9-spirobifluorene) (spiro-MeOTAD), 28.8 μL 4-tert-butylpyridine, 17.5 μL of a stock solution of 520 mg/mL lithium bis(trifluoromethylsulphonyl)imide in acetonitrile, and 29 μL of a solution of 300 mg/mL FK209 in acetonitrile in 1 mL chlorobenzene.

### Preparation

The indium tin oxide (ITO) glasses were cleaned sequentially in acetone, absolute ethyl alcohol, and deionized water ultrasonic bath for 15 min, respectively. After ITO glasses were cleaned by the UV-ozone treat for 20 min, a SnO_2_ film was deposited by spin-coating diluted SnO_2_ nanoparticle colloidal solution (Alfa Aesar (tin(IV) oxide, 15% in H_2_O colloidal dispersion)) according to reference [31]. After the spin-coating, the SnO_2_ film was heated at 165 °f for 0.5 h. Then the substrates were treated with the UV-ozone again and transferred into the glovebox. Perovskite films were prepared by spin-coating with a speed of 1000 rpm for 10 s and 5000 rpm for 45 s. At 9 s before the ending of the spin-coating program, 150 μL chlorobenzene was dropped onto the spinning substrate. Then, the perovskite films were heated at 100 °C for 60 min. The HTL was prepared by spin-coating the HTL solution at 5000 rpm for 30 s. Finally, 100 nm of Au top electrode was thermally evaporated onto the HTL.

### Characterization

The current density-voltage (J-V) characteristic of PSCs was recorded by Keithley source unit 2400 under AM 1.54G sun intensity illumination by a solar simulator from Newport Corp. The X-ray diffraction patterns were recorded with Bruker D8 ADVANCE A25X. Fourier-transform infrared spectroscopy (FTIR) and scanning electron microscope (SEM) were conducted on Nicolet iS10 and field emission fitting SEM (FEI-Inspect F50, Holland). The absorption of perovskite was measured using Shimadzu 1500 spectrophotometer.

## Results and Discussion

**Fig. 1:**
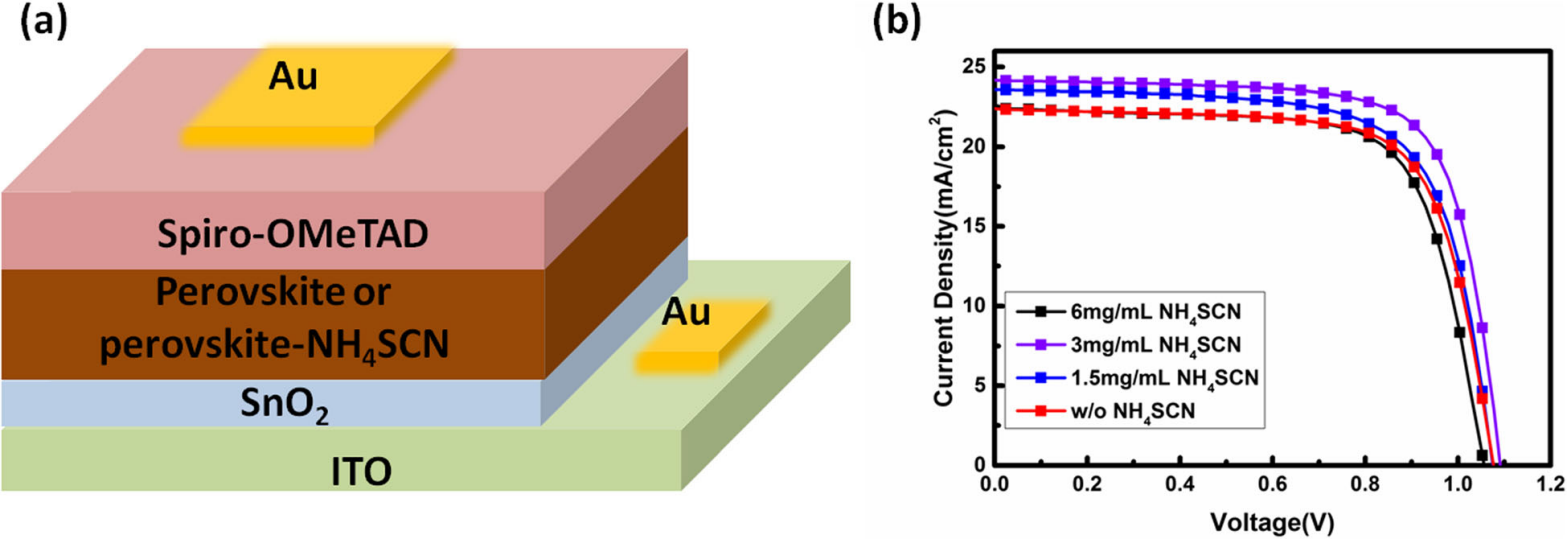
**a** Schematic illustration of PSCs structure. **b** J-V curves of PSCs based on perovskite films deposited from perovskite precursors doped with different NH_4_SCN

**Fig. 2:**
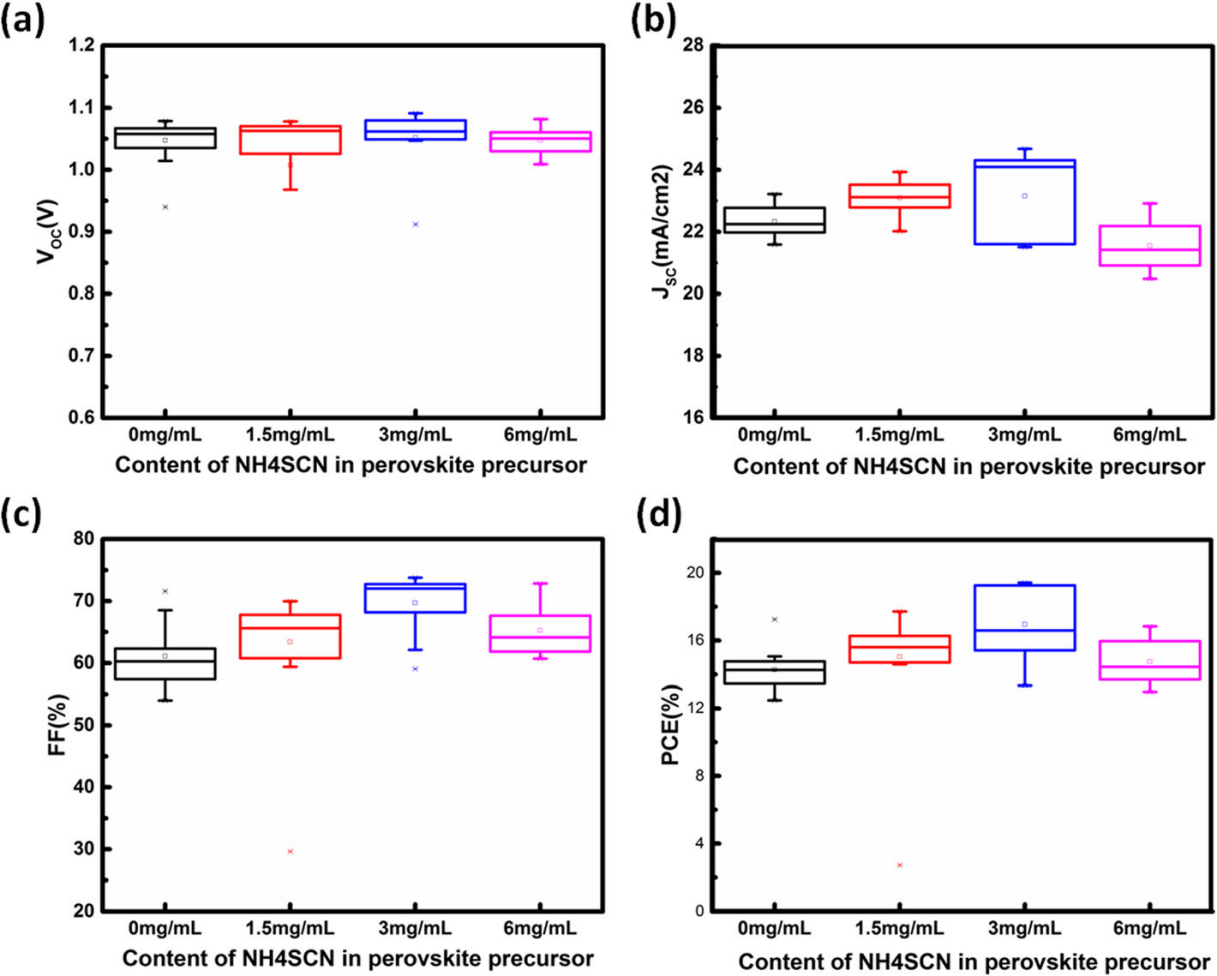
**a**–**d** Statistical data for V_OC_ (**a**), J_SC_ (**b**), FF (**c**), and PCE (**d**) of PSCs based on perovskite films deposited from perovskite precursors doped with different NH_4_SCN

**Fig. 3:**
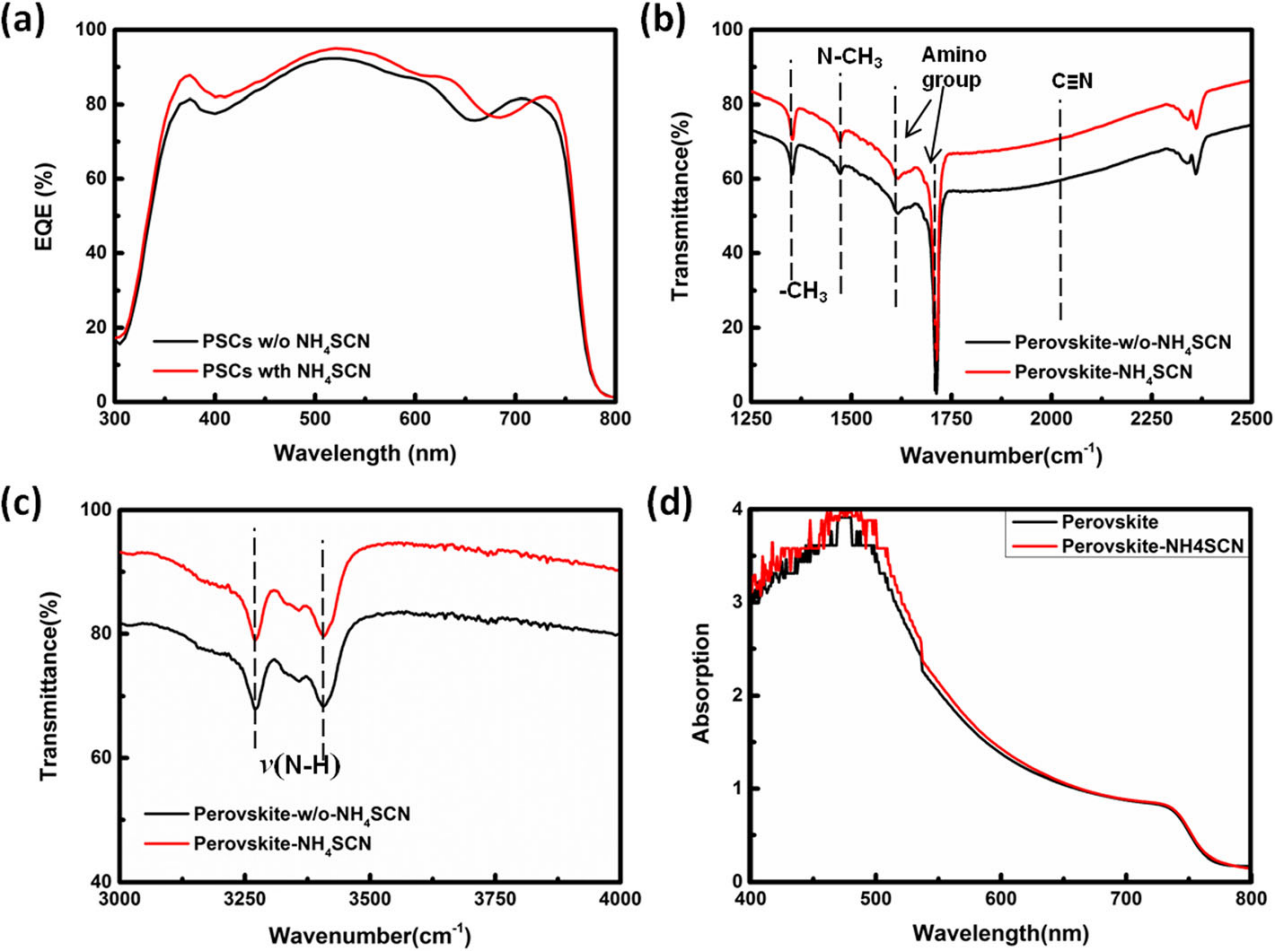
**a** EQE spectrum of target PSCs (PSCs with NH_4_SCN) and reference PSCs (PSCs w/o NH_4_SCN). **b**, **c** FTIR results of the perovskite-w/o-NH_4_SCN films and perovskite-NH_4_SCN films. **d** UV-vis absorption spectrum of the perovskite-w/o-NH_4_SCN films and perovskite-NH_4_SCN films

To optimize the content of NH_4_SCN, perovskite films were deposited using the perovskite precursor solutions doped with different content of NH_4_SCN, and these films were used as the light harvester layers of solar cells. The configuration of PSCs is ITO/SnO_2_/perovskite/Spiro-OMeTAD/Au, as shown in Fig. 1a. To simplify the expression in this article, the perovskite film fabricated from perovskite precursor solutions doped with a concentration of *x* mg/mL is expressed as perovskite-*x* here. The current density-voltage (J-V) curve of the champion device in each group is plotted in Fig. 1b, and the corresponding photovoltaic parameters are listed in Table 1. The statistical data for photovoltaic parameters of PSCs are shown in Fig. 2a–d. The perovskite-1.5-based PSCs (target PSCs) exhibit the best photovoltaic performance, attributed to the improved short-circuit current density (J_SC_) and fill factor (FF). Compared with the champion perovskite-0-based PSCs (reference PSCs), all the photovoltaic parameters of the champion PSCs based on perovskite-3 have been enhanced obviously, resulting in a PCE of 19.36%. The external quantum efficiency (EQE) spectrums of target PSCs and reference PSCs are shown in Fig. 3a. The EQE values of target PSCs at most of the visible light region are higher than that of reference PSCs, consisting with the EQE result in reference [26]. This phenomenon results from the more efficient charge transport in perovskite films with better crystallinity. To investigate the mechanism for the photovoltaic performance improvement, several characterizations have been carried out on the perovskite films.

**Table 1:** Photovoltaic parameters of the champion device in each group

Device	J_SC_ (mA/cm^2^)	V_OC_ (V)	FF (%)	PCE (%)
Perovskite-0-based PSCs	22.37	1.077	71.58	17.24
Perovskite-1.5-based PSCs	23.57	1.075	69.88	17.71
Perovskite-3-based PSCs	24.17	1.091	73.39	19.36
Perovskite-6-based PSCs	22.45	1.056	71.04	16.85

The reflecting FTIR measurement has been carried on perovskite films without NH_4_SCN doping (perovskite-w/o-NH_4_SCN) and perovskite films with NH_4_SCN doping (perovskite-NH_4_SCN) to identify the organic groups and ingredients in perovskite films, as shown in Fig. 3a. The absorption peaks at the wavenumber of 1350 cm^−1^ and 1477 cm^−1^ are attributed to the vibration of organic –CH_3_ groups in perovskite films. The corresponding absorption peaks of the amino groups in perovskite films locate at the range of 1600–1750 cm^−1^ and 3200–3500 cm^−1^. No absorption peak corresponding to –C≡N in –SCN can be found in perovskite-NH_4_SCN, demonstrating that there is no residue of NH_4_SCN in the final perovskite-NH_4_SCN films. The UV-visible light absorption also has been measured and the result is shown in Fig. 3b. Both perovskite films have a strong absorption when the light wavelength is below 750 nm, and the absorption edges of both perovskite films overlap, clarifying the bandgap values of both perovskite films are the same. The similar shape of the FTIR plots and UV-visible absorption curves of the perovskite-w/o-NH_4_SCN and perovskite-NH_4_SCN indicates that both perovskite films have the same ingredient.

**Fig. 4:**
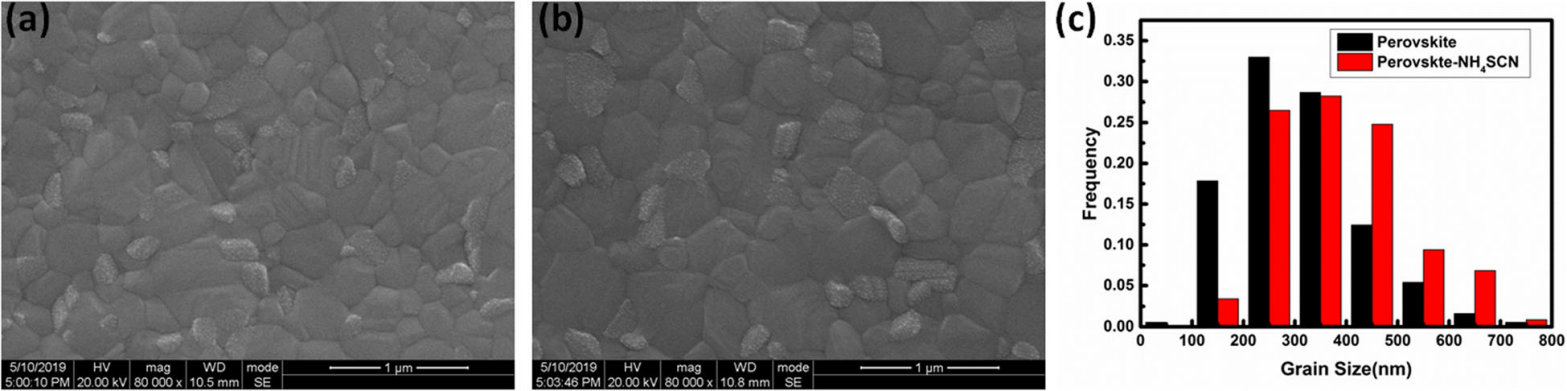
**a**, **b** Surface SEM image of perovskite-w/o-NH_4_SCN films (**a**) and perovskite-NH_4_SCN films (**b**). **c** Histogram for grain-size distribution of the crystals in surface SEM images

The morphology of perovskite films is investigated using SEM, and the results are shown in Fig. 4a, b. The perovskite films without NH_4_SCN dopants contain many small-size crystals with a gain size lower than 200 nm. In contrast, there are much fewer small-size crystals in perovskite-NH_4_SCN films. The average crystal grain sizes of both perovskite films have been calculated using the Nano Measurer software. The average crystal grain size of perovskite-w/o-NH_4_SCN and perovskite-NH_4_SCN is about 312.02 nm and 382.95 nm, respectively. The grain-size distributions of the crystals in SEM images are shown in Fig. 4c. The frequency of the crystal grain size distributed at the range of 200–300 nm is highest in perovskite-w/o-NH_4_SCN. However, the frequency of the crystal grain size distributed at the range of 300–400 nm is highest in perovskite-NH_4_SCN. The distribution proportion of the grain size larger than 400 nm in perovskite-w/o-NH_4_SCN is also much lower than that in perovskite-NH_4_SCN. The larger grain size in perovskite results in the fewer crystal grain boundaries. It has been reported that the trap states are mainly distributed on the boundaries of perovskite crystal grains. Hence, the perovskite-NH_4_SCN films with larger-size crystal grains are favored by high-performance PSCs.

The X-ray diffraction (XRD) pattern has been used to identify the crystallinity of perovskite films further. There are no distinct peak location shifts in the XRD pattern of perovskite-NH_4_SCN films compared with the XRD pattern of perovskite-w/o-NH_4_SCN films, demonstrating that both perovskite films show the same crystallization type. The peaks at 14.37°, 20.27°, 24.82°, 28.66°, 32.12°, 35.38°, 40.88°, and 43.46° correspond to (001), (011), (111), (002), (012), (112), (022) and (003) planes of perovskite films, respectively. The peaks at 12.93° originate from the PbI_2_ crystal grains. The strongest peak in XRD patterns locates at 14.37°, so we have magnified the XRD patterns at a range of 12–15° to observe the crystallinity difference between these two perovskite films accurately. The PbI_2_ peak intensity in the XRD pattern of the perovskite-NH_4_SCN is lower than that of the perovskite-w/o-NH_4_SCN, indicating that less PbI_2_ byproduct can be observed. Except the excess PbI_2_ in perovskite precursor, PbI_2_ can also be generated when the perovskite is annealed due to the escape of some organic cation salts. Hence, it can be inferred that perovskite-NH_4_SCN films show better thermal stability. The (001) plane peak intensity in the XRD pattern of perovskite-NH_4_SCN films is higher than that of perovskite-w/o-NH_4_SCN, and the (001) plane peak width at half the height in the XRD pattern of perovskite-NH_4_SCN films is much smaller, clarifying perovskite-NH_4_SCN films show better crystallinity.

**Fig. 5:**
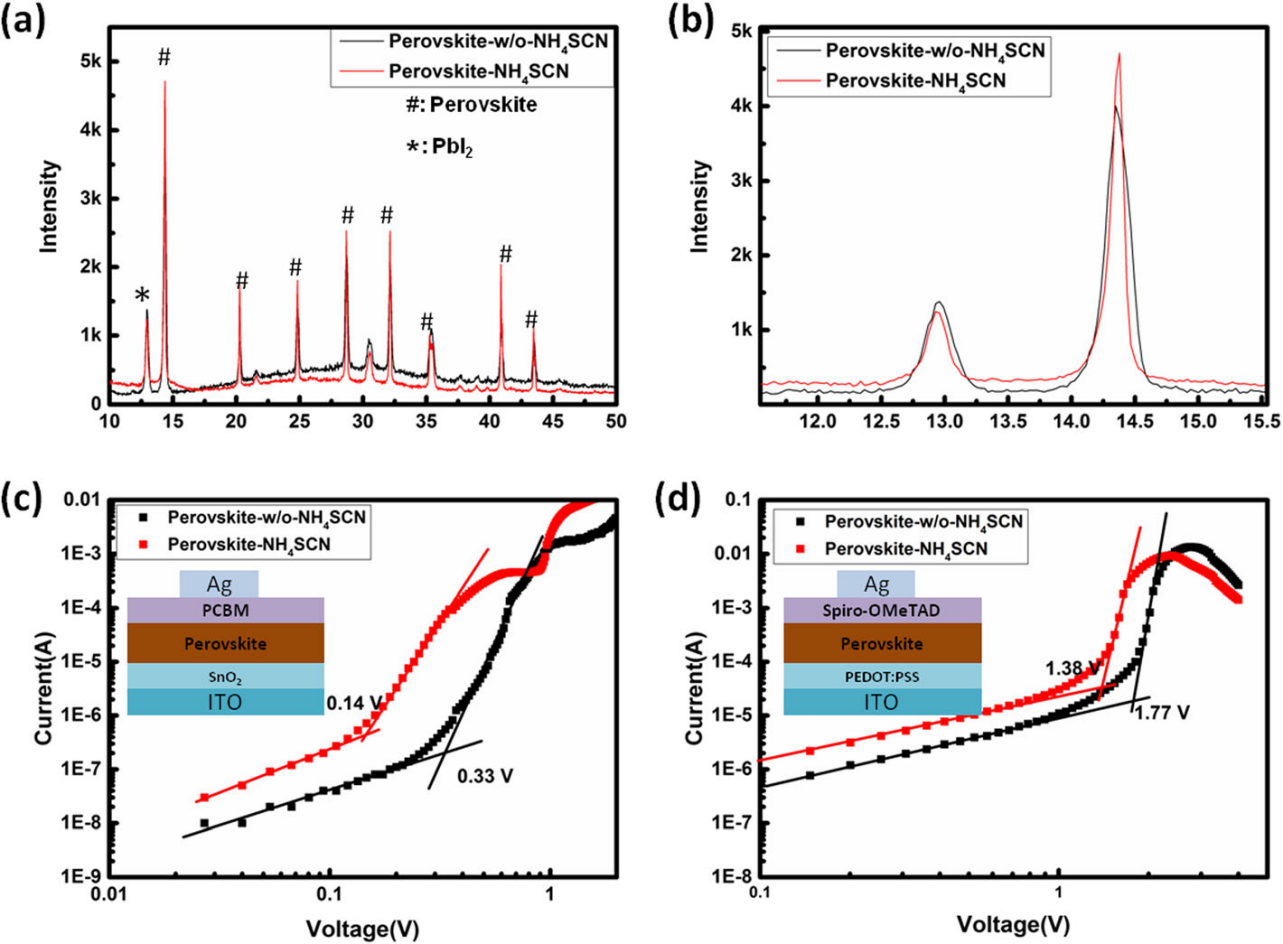
**a** XRD patterns of perovskite-w/o-NH_4_SCN films and perovskite-NH_4_SCN films. **b** Magnified XRD patterns at a range of 12–15°. **c** dark I-V curves for electron-only devices based on different perovskite films (inset: configuration of electron-only devices). **d** Dark I-V curves for hole-only devices based on different perovskite films (inset: configuration of hole-only devices)

The electron-only devices and hole-only devices have been fabricated to characterize the electron trap-state density and hole trap-state density in both perovskite films, respectively. The configuration of electron-only devices and hole-only devices is presented in the inset of Fig. 5 c and d, respectively. The dark current-voltage (I-V) curves of the devices have been measured and plotted in Fig. 5c, d. All I-V curves contain ohmic response region at low bias voltage region. As the voltage is continuously increased, the current rises steeply due to the reduced trap density. The kink point (*V*_TFL_) of these curves can be used to identify the trap-state density according to equation (1) [32–35]:

1 $$ {V}_{\mathrm{TFL}}=\frac{\mathrm{e}{\mathrm{n}}_{\mathrm{t}}{\mathrm{L}}^2}{2\upvarepsilon {\upvarepsilon}_0} $$

where *L* is the thickness of the perovskite films, ε is the relative dielectric constant of perovskite films, *n*_*t*_ is the trap-state density, and ε_0_ is the vacuum permittivity. The similar absorption intensity shown in Fig. 3b indicated that the thickness of both perovskite films is very close. The FTIR results and UV-visible light absorption edges show that the ingredients in perovskite films are the same. Therefore, both perovskite films have the same ε value. The *V*_TFL_ is relative to the trap-state density positively. As shown in Fig. 5c, d, both *V*_TFL_ values obtained from perovskite-NH_4_SCN-based electron-only devices and perovskite-NH_4_SCN-based hole-only devices are obviously lower than those obtained from perovskite-w/o-NH_4_SCN-based electron-only devices and perovskite-w/o-NH_4_SCN-based hole-only devices. It clarifies that both electron trap-state density and hole trap-state density have been reduced in perovskite-NH_4_SCN films due to the increased crystal grain size.

From the above characterization and analysis, we can infer that using perovskite precursor solution doped with NH_4_SCN to deposit perovskite films increases the perovskite crystal grain size, resulting in a reduced crystal boundary area and trap-state density. The reduced trap-state density in perovskite is beneficial to the enhanced charge transport and photovoltaic performance of PSCs.

## Conclusion

In conclusion, we have adapted NH_4_SCN as the dopant of perovskite precursors to increase the crystalline of perovskite films. The enhanced-crystallinity perovskite-based PSCs achieve the champion PCE of 19.36% which is much higher than the maximum PCE of the reference PSCs (17.24%). The improved photovoltaic performance of target PSCs is attributed to the enhanced crystal grain size in perovskite-NH_4_SCN films. The enhanced crystal grain size in perovskite-NH_4_SCN films can reduce the charge trap-state density and benefit to the charge transport. Our results demonstrate a simple and effective way to enhance the device efficiency by improving the crystalline of perovskite films.

## Data Availability

All the data are fully available without restrictions.

## Abbreviations

DMF: Dimethylformamide
DMSO: Dimethyl sulfoxide
ETL: Electron transport layer
FA: HC(NH_2_)_2_
FF: Fill factor
FTIR: Fourier-transform infrared spectroscopy
HTL: Hole-transport layer
ITO: Indium tin oxide
J_SC_: Short-circuit current density
J-V: Current density-voltage
MA: CH_3_NH_3_
NH_4_SCN: Ammonium thiocyanate
OIMHP: Organic-inorganic hybrid metal halide perovskite
PCE: Power conversion efficiency
PMMA: Poly(methyl methacrylate)
PSCs: Perovskite solar cells
SEM: Scanning electron microscope
Spiro-OMeTAD: (2,29,7,79-tetrakis(*N*,*N*-di-p-methoxyphenylamine)-9,9-spirobifluorene)
TS-QLE: Theoretical Shockley–Queisser limit efficiency
V_OC_: Open-circuit voltage
XRD: X-ray diffraction
